# Oral Vaccines: A Better Future of Immunization

**DOI:** 10.3390/vaccines11071232

**Published:** 2023-07-12

**Authors:** Keith Wai-Yeung Kwong, Ying Xin, Nelson Cheuk-Yin Lai, Johnny Chun-Chau Sung, Kam-Chau Wu, Yusuf Khwaja Hamied, Eric Tung-Po Sze, Dominic Man-Kit Lam

**Affiliations:** 1 Research Department, DreamTec Cytokines Limited, Hong Kong, China; xinying@dreamtec.hk (Y.X.); nelsonlai@dreamtec.hk (N.C.-Y.L.); johnnysung@dreamtec.hk (J.C.-C.S.); kcwu@dreamtec.hk (K.-C.W.); 2 Oristry BioTech (HK) Limited, Hong Kong, China; 3 Theratide BioTech (HK) Limited, Hong Kong, China; 4 Cipla Limited, Mumbai 400013, India; ykh@cipla.com; 5 School of Science and Technology, Hong Kong Metropolitan University, Hong Kong, China; esze@hkmu.edu.hk; 6 DrD Novel Vaccines Limited, Hong Kong, China; dlam@worldeye.org; 7 Torsten Wiesel International Research Institute, Sichuan University, Chengdu 610064, China

**Keywords:** oral vaccine, gene therapy, immunity

## Abstract

Oral vaccines are gaining more attention due to their ease of administration, lower invasiveness, generally greater safety, and lower cost than injectable vaccines. This review introduces certified oral vaccines for adenovirus, recombinant protein-based, and transgenic plant-based oral vaccines, and their mechanisms for inducing an immune response. Procedures for regulatory approval and clinical trials of injectable and oral vaccines are also covered. Challenges such as instability and reduced efficacy in low-income countries associated with oral vaccines are discussed, as well as recent developments, such as Bacillus-subtilis-based and nanoparticle-based delivery systems that have the potential to improve the effectiveness of oral vaccines.

## 1. Introduction

The development of vaccines was one of the most important breakthroughs in healthcare and medicine. Preceding Edward Jenner’s creation of the smallpox vaccine [1], infectious diseases had killed countless people. Since then, vaccines have had a profound impact, saving millions of lives by eradicating the spread of devastating diseases such as polio [2], measles, and diphtheria [3]. As a result, vaccines have been considered among the most effective public health interventions.

Despite being efficient at preventing diseases, conventional vaccine delivery techniques using injections through intramuscular and intravenous routes also come with a variety of drawbacks [4], for example, requiring qualified medical staff, storage of vaccines at low temperatures, and pain at the injection site [5,6]. Researchers have focused on the creation of alternate, less invasive vaccine delivery systems, such as oral vaccinations, to overcome these issues [7,8,9,10].

A milestone in vaccine history occurred with the introduction of the first oral vaccinations in the 1960s; the oral polio vaccine (OPV) was applied in the fight to eradicate polio [11]. After the success of OPV, numerous oral vaccines were developed and proven to be effective in preventing diseases including cholera, rotavirus, and typhoid [12,13,14,15]. With administration into the gut directly, the primary target of oral vaccines would be the gut-associated lymphoid tissue (GALT), a crucial region for vaccination-induced immune responses [16]. The ease of administration and simple manufacturing procedures are also advantages of oral vaccine administration over conventional injection-type vaccines [17] (Table 1). Moreover, oral vaccinations are a desirable alternative for individuals with weakened immune systems, because the blood vessels and circulatory system are avoided [18,19].

Although there have been significant advancements in the development of oral vaccines recently, there are still numerous obstacles that must be overcome in order to fully harness their potential [20,21]. This review aspired to deliver a comprehensive analysis of the present status of oral vaccines, and to delve into the potential opportunities and impediments linked to their advancement and implementation.

## 2. The Mechanism of Inactivated Virus/Bacteria Oral Vaccines

Oral vaccines function differently than other vaccine routes, allowing the gut-based mucosal immune system to be exposed to antigens, such as inactivated viruses or bacteria, and provoke a localized response specifically in the gut, the primary entry site for many pathogens [22]. The antigens of oral vaccines then decline due to degradation in the stomach and intestine [23].

The antigen uptake by gut-dwelling antigen-presenting cells, such as dendritic cells or specialized M cells, launches the transcytosis process, with M cells playing a vital role in antigen capture and subsequent presentation to the underlying T cells, which act as a master controller in guiding the immune response [24]. Upon recognizing the antigen, T cells activate and differentiate into effector T cells. These cells then migrate to the site of infection and release cytokines, which stimulate the production and differentiation of other immune cells, such as B cells. The B cells subsequently mature into plasma cells, which generate IgA and IgG to evoke mucosal and systemic immune responses. T helper cells facilitate B cell differentiation into memory B cells for memory responses [25]. In addition, activated T cells neutralize pathogens directly while recruiting other immune cells, including natural killer cells and macrophages, assisting pathogen destruction [26]. The gut mucosa, saturated with native bacteria and particular immune cells, is the primary location for oral vaccines to trigger the immune response [27]. The GALT contains a significant number of immune cells, including T cells, B cells, and plasma cells, which provide mucosal immunity [28]. Peyer’s patches, a collection of lymphoid follicles inside the small intestine’s epithelium, identify antigens from pathogens, symbiotic bacteria, and food proteins in order to enable immune cells to generate a response to these antigens and protect the individual from infection, while instilling tolerance [22].

### The Dominance of Secretory IgA in the Oral Vaccine-Induced Immune Response

The production of secretory Immunoglobulin A (secretory IgA) in GALT is especially notable in an orally induced immune response. Secretory IgA can block pathogens located on mucosal surfaces such as the gut through the secretion of secretory components into the lumen of the gut. Conversely, when the immune system is activated by subcutaneous or intramuscular injection, Immunoglobulin G (IgG) is predominantly created, which is distributed through the bloodstream to protect against invading organisms systemically [29].

**Figure 1:**
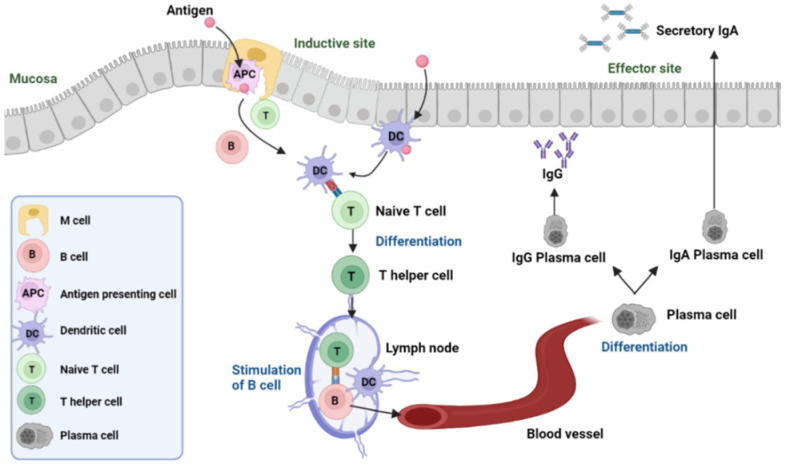
Schematic diagram outlining the immune responses in the intestine. At the inductive site, M cells transfer the antigen to the antigen-presenting cells. Antigen-presenting dendritic cells (DCs) facilitate naive T cells to differentiate into T helper cells. The activated T cells stimulate B cells, which further leave the lymph nodes to enter the circulatory system. B cells migrate to effector sites and differentiate into plasma cells, which in turn produce IgA and IgG.

Secretory IgA is generated by the administration of oral vaccinations as a targeted response within the gut, the usual site of exposure to pathogens. The involvement of M cells, a type of epithelial cell, along with other immune cells found in the GALT, allows antigens to be taken up easily, and promotes an effective immune response. On the other hand, with injection vaccines, a generalized response is evoked and there is a lower output of IgG production (Figure 1).

Considering that pathogens frequently access the body through mucosal surfaces such as the respiratory tract, oral vaccines are an ideal choice due to the heightened production of secretory IgA. Secretory IgA is most commonly found in mucous secretions such as tears, saliva, and mucus, and is a necessary element for a fully functioning mucosal immunity system. Systemic immunity relies mainly on IgG, but mucosal immunity acts as the initial defense mechanism at mucosal sites, warding off harmful microbes before they can reach the other areas of the body [30].

## 3. Current Developed and Certificated Oral Vaccines

Immunization via oral vaccines is a form of delivery distinct from injection, and these vaccines can be classified as an OPV, oral cholera vaccine (OCV), oral rotavirus vaccine (ORV), or oral typhoid vaccine (OTV). Current licensed oral vaccines are summarized in Table 2. In addition to the use of inactivated virus/bacteria-based oral vaccines, the immune response can also be provoked orally by adenovirus-based vaccines, plant-based vaccines, and recombinant protein-based vaccines.

Adenovirus-based oral vaccines utilize replication-deficient adenoviruses to transport the antigen directly to the gut mucosa [42]. In contrast, some specific recombinant protein-based vaccines link the antigen to an adjuvant to cause an immune response in the gut [43]. Plant-based vaccines, on the other hand, use edible plants to transfer the antigen to the body [44].

Every vaccine above comes with its own unique pros and cons, which will be outlined in the sections below.

### 3.1. Inactivated Virus/Bacteria Oral Vaccine

The OPV was the world’s first oral vaccine, developed by Jonas Salk in the 1950s, and is still used in many developing nations. After gaining approval for use in the United States in 1963, OPV rapidly became the primary polio vaccine in the country’s immunization program during the mid-1960s [11]. Companies such as Sanofi Pasteur and GlaxoSmithKline produce OPV, which is a live but weakened viral vaccine containing inactivated virus particles unable to cause illness. As it has the potential to cause vaccine-associated paralytic poliomyelitis (VAPP), with a probability of approximately 1 in 2.4 million doses [45], an injection-type inactivated poliovirus vaccine (IPV) has been used to replace OPV in several countries [46].

An OCV is a live, weakened vaccine, which has been utilized since the 1990s [47] to stave off cholera, a disease caused by the bacterium Vibrio cholera. This vaccine, which is produced by various companies including Shantha Biotechnics and Eubiologics, has been prequalified by the World Health Organization for use in regions with high cholera rates, including India, Bangladesh, Pakistan, and Haiti. Despite being effective, an OCV only provides temporary protection and requires booster doses to maintain immunity. Additionally, OCVs have been associated with side effects, such as diarrhea and nausea [48].

The ORV used to protect against rotavirus, a leading cause of serious diarrhea in babies and young children, was developed in the 1990s and is manufactured by a variety of companies, such as Merck and GSK [49]. ORVs are relatively inexpensive and easy to administer, and give protection within a few days after vaccination [50]. There are two types of ORV available: namely, a live, attenuated vaccine and an inactivated vaccine. The live, attenuated vaccine offers better protection, but there is a chance of viral shedding which therefore may put immunocompromised individuals at risk. On the other hand, the killed vaccine does not cause shedding, but does not provide as much protection [51].

The OTV is utilized to avert typhoid fever, a bacterial infection caused by Salmonella typhi [41]; it created in the 1990s and is manufactured by various companies, such as Bharat Biotech and Crucell. OTVs are a low-cost and easy-to-access vaccine that can protect against typhoid within a few days after vaccination. Similar to OCVs, however, they only provide short-term protection and multiple booster doses are required to sustain immunity [52].

### 3.2. Adenovirus Oral Vaccines

**Figure 2:**
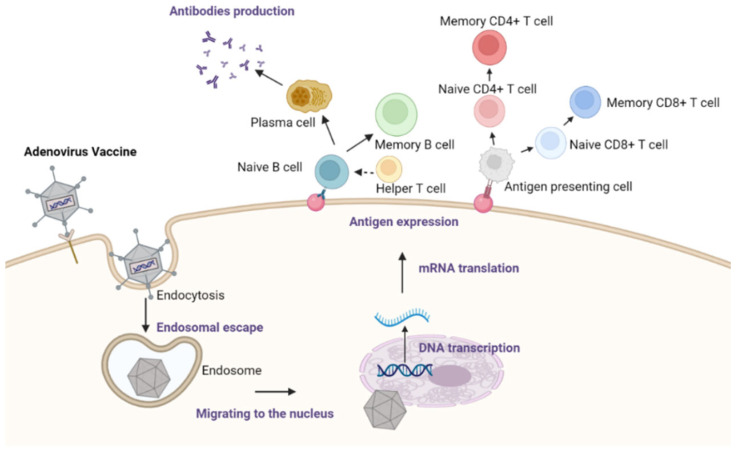
The mechanism of adenoviral vaccine-induced immune responses. The Adenovirus vaccine is taken up through endocytosis, followed by escape from the endosome. After migrating to the nucleus through the microtubule, the vaccine transgene antigen is transcribed. Then, the corresponding protein is translated from mRNA and the antigen is expressed on the membrane. Helper T cells facilitate B cells to differentiate into antibody-secreting plasma cells and generate antibodies, along with memory B and T cells.

Adenoviruses are a group of viruses that belong to the Adenoviridae family. They can infect different organisms, including humans, animals, and birds. These viruses contain a linear double-stranded DNA genome protected by an icosahedral protein shell known as a capsid. The capsid structure enables the viruses to enter the target cells and replicate their genome to produce the target antigen [53,54] (Figure 2).

Molecular engineered adenoviruses have been employed as vectors for gene therapy and oral vaccines, providing cost-effective solutions to the delivery of genes or vaccine antigens to the target cells efficiently and effectively. They can be produced in large quantities, and they are able to express multiple genes or antigens. Moreover, their safety can be enhanced by the use of replication-defective or non-replicating adenoviral vectors. Therefore, adenoviruses are ideal for oral-based vaccine delivery systems, especially to infect various gut cells, including intestinal epithelial cells and antigen-presenting cells, through direct delivery of vaccine antigens to the GALT to trigger mucosal immunity [55].

Serotypes refer to the distinct variations of a microorganism or virus that possess different surface antigens [56]. Among the various adenoviruses, human adenovirus serotypes 4 (HAdV-4), 5 (HAdV-5), and 7 (HAdV-7) are commonly used as oral vaccines [42,57]. These serotypes are attractive due to their efficient infection of the GALT. For example, a recombinant HAdV-5 expressing the spike protein of SARS-CoV-2 has been developed as an oral vaccine against COVID-19 [57]. Preclinical studies in hamsters have also shown that oral administration of this vaccine induces a strong immune response and provides protection against SARS-CoV-2 infection.

Another example of an adenovirus-based oral vaccine applies to live attenuated adenovirus to express cholera toxin B subunit (CTB) against cholera. Preclinical studies have shown that oral administration of this vaccine induces a strong immune response and further provides robust protection against cholera. Clinical trials have also assessed the safety and efficacy of this vaccine in humans, with promising results thus far [9].

### 3.3. Recombinant Protein-Based Oral Vaccines

Recombinant protein-based oral vaccines employ genetically engineered proteins or peptides to trigger a robust immune response and provoke a protective response against various diseases, including influenza [58], nervous necrosis virus [59], and SARS-CoV-2 [60]. These vaccines are produced by expressing the targeted antigen from a suitable recombinant expression system, such as bacteria, yeast, or mammalian cells, followed by purification and formulation for vaccine production [61,62,63]. Recombinant protein vaccines can be administered through either the injectable or oral route; for example, recombinant protein-based vaccines include human papillomavirus (HPV) vaccines Gardasil 9 and Cervarix [64], the hepatitis B vaccine Engerix-B [65,66], and the respiratory syncytial virus (RSV) vaccine Synagis [67]. These vaccines have demonstrated their efficacy in protecting against their respective diseases.

Adjuvants are a crucial factor in enhancing the efficacy of recombinant protein-based oral vaccines. Adjuvants are compounds that increase the immune system’s response to the antigen, which results in a stronger and more sustained immunity. This is important where the antigen lacks substantial immunogenicity or is metabolized rapidly in the body [68,69].

Various forms of adjuvants, including cholera toxin, chitosan/aluminum, and glucan, are commonly utilized in recombinant protein-based oral vaccines, and are well-known for their cost-effectiveness and safety [70,71,72]. Another example is a squalene-based adjuvant to elicit strong immune responses by sustained release of antigens in the body [73].

The choice of adjuvant for a vaccine is contingent upon several factors, including the stability of the antigen, desired immune response, intended administration route, and safety concerns. The suitability of an adjuvant for the target vaccine can be determined through a combination of preclinical and clinical studies. With the use of adjuvants, recombinant protein-based oral vaccines can provide enhanced protection against diseases and make a vital contribution to public health initiatives.

### 3.4. Transgenic Plant-Based Oral Vaccines

Transgenic plants are an emerging and innovative platform for recombinant protein production due to their various advantages [74,75]. Significant quantities of protein can be produced in a cost-effective manner by altering the plant’s genome genetically to express a desired recombinant protein [76,77]. The use of transgenic plants for the production of oral vaccines is particularly noteworthy, as they offer a safe and reliable option for inducing an immune response [78].

The safety of transgenic plant-based oral vaccines is their primary benefit, due to the inherent defense mechanisms of plants, to protect against infections and minimize the risk of contamination [79]. Moreover, the absence of animal-derived materials in the recombinant protein production process reduces the risk of transmitting animal-borne diseases. The use of a plant model also provides an ethical option, without any animal origins [80].

The use of transgenic plants for recombinant protein production also provides a highly controlled and monitored environment, reducing the potential for contamination by pathogens [76,81]. Furthermore, the large-scale production capacity of transgenic plants makes them ideal for emergency vaccine production, which is essential in the event of a pandemic health crisis [82].

Studies in mice have demonstrated the efficacy of transgenic plant-based oral vaccines, such as one produced using HBsAg-transgenic potatoes [83]. This has led to increasing interest in the production of oral vaccines and recombinant proteins via the use of transgenic plants. As a result of these benefits, the use of transgenic plants for the production of recombinant proteins and oral vaccines is a highly promising area of research and development.

## 4. Regulatory Procedures and Clinical Trials for Injectable Vaccines vs. Oral Vaccines

The approval and authorization procedures for oral and injectable vaccines are quite alike, but there are some differences. Prior to being licensed for use by regulatory agencies such as the U.S. Food and Drug Administration (FDA), the National Medical Products Administration (NMPA) of China, or the European Medicines Agency (EMA), both oral and injectable vaccines must go through strenuous preclinical and clinical trials to demonstrate their safety, efficacy, and stability [84]. During the preclinical phase, in vitro and in vivo models are used to determine the safety and efficacy of vaccine candidates, and to decide the starting dose and administering schedule for clinical studies [85].

Variations in dosage must be considered when developing oral and injectable vaccines. Injectable vaccines are regularly administered in one or multiple doses, and the number of doses administered is contingent upon the vaccine being given, as well as the person’s age, weight, and immune system [86,87]. Conversely, oral vaccines need extra testing to investigate their resistance in the acidic milieu of the stomach, as well as their ability to stay intact during the digestive process and their power to activate an immune reaction [27]. Usually, injectable vaccines are directly given into the muscle or bloodstream, thus making a more compact dose adequate. Oral vaccines usually require a higher dosage due to loss during digestion, and to ensure an adequate amount of vaccine arrives at the target site for the immune response to occur [88]. The starting dose of an oral vaccine candidate determined from in vitro and in vivo studies during the preclinical phase may not be applicable to clinical studies due to the low recovery of the vaccine after digestion. In addition, the formulation of the vaccine, including other ingredients, may affect the final amount of vaccine at the target cells as well. Application of adjuvants or other delivery systems may increase their efficacy and reduce the amount of vaccine required for both oral-based [89] and injection-based vaccines [69]. Vaccine bioavailability refers to the amount of the vaccine that is absorbed and available for use by the body after administration. Generally, injected vaccines have high bioavailability because they are directly injected into the bloodstream, bypassing the digestive system. Oral vaccines, on the other hand, have lower bioavailability because they must pass through the digestive system before being absorbed. Nanovesicles can be adopted to enhance the bioavailability of oral vaccines [90,91].

Safety is a vital consideration during pre-clinical and clinical studies, and the approval processes of national pharmaceutical agencies. Injectable vaccines are normally given through intramuscular or subcutaneous shots and their security profile depends greatly on the antigens and adjuvants utilized in the vaccine [92]. The most widely reported adverse reactions related to injectable vaccines are local responses, such as pain, swelling, and redness at the injection site, and systemic effects such as fever, malaise, and headaches. The risk of undesirable effects is dependent on numerous factors, including the vaccine ingredients, the person’s vulnerability status, and past medical history [93]. Local side effects are extremely common among injected vaccines. For participants aged 18–30, the prevalence of injection site pain, swelling, and redness is 91.8%, 17.4%, and 13.4%, respectively, after receiving COVID-19 vaccines [94]. Similarly, the incidence of local side effects is 83.1% in the intradermally fractionated dose poliovirus vaccine group and 59.8% in the intramuscular full dose poliovirus vaccine group [95]. However, injection site reactions can be avoided through oral vaccine administration. Participants may exhibit systemic side reactions after receiving oral and injection vaccines. For example, the injectable COVID-19 vaccines with messenger RNA (mRNA) technologies from Pfizer-BioNTech and Moderna utilize the recipients’ cells to produce a segment of the spike protein found on the surface of the SARS-CoV-2 virus, thereby prompting an immune response and helping to protect against the viral infection [96,97]. Despite the fact that these vaccines have indicated high efficacy in producing antibodies, some people may experience various symptoms identified from the immune reaction activated by the vaccine, including pain and swelling at the injection site, exhaustion, headache, muscle pain, chills, fever, and nausea [98]. Some individuals have reported uncommon blood clot events after the vaccination [99,100], particularly among young women; however, the exact mechanism for this effect is still unknown [101]. It is speculated that the translation of spikes from mRNA vaccines may trigger an immune response that leads to clotting, prompting genuine reactions such as stroke, deep vein thrombosis (DVT), pulmonary embolism, myocarditis, and acute hepatitis [102].

Oral vaccines have been developed to generate an immune response through an atypical way of administration. Unlike injectable vaccines that need to enter the bloodstream, oral vaccines are consumed and recognized by immune cells along the GALT of the digestive system, which has a significant role in the processing and introduction of antigens to the immune system [16]. As a result, adjuvants may not be necessary for oral vaccine formulations to initiate an immune response, whereas they are generally obligatory for injectable vaccines to fortify the immune response.

The digestive tract is an ideal setting for the triggering of immune reactions due to its broad surface area and large population of immune cells. By exploiting the natural handling and exhibition of antigens by digestive system-residing immune cells, oral vaccines can trigger a potent immune response [103].

Even though some oral vaccines (e.g., OPV) may have mild side effects, such as headache, fever, vomiting, and diarrhea, the effects are generally minor and transient [18,104]. In rare cases, vaccine-associated paralytic polio has been reported following the administration of OPV [105]. Considering factors such as ease of administration and the avoidance of invasive needle injections, oral vaccines are a favorable option for many applications, especially in circumstances where needle-based delivery is not feasible [48].

Oral vaccines can be cost-effective in certain situations. For example, the injectable polio vaccine is much more expensive than the oral polio vaccine. The price of the IPV ranges from USD 1.00 to USD 3.28 per dose, while the price of the OPV is USD 0.12–USD 0.18 per dose [105]. Although the majority of countries offer vaccines to their citizens free of charge, the vaccination rate in impoverished countries is low because they cannot afford to purchase enough vaccines [106]. As low-income countries encounter difficulties accessing, delivering, and utilizing vaccines, oral vaccines could help to overcome the above obstacles. From a developmental cost perspective, oral vaccines are an attractive choice for small business owners who have limited resources [27].

### Cost of Clinical Trials: Injectable Vaccines vs. Oral Vaccines

The disparity in the cost of clinical trials and regulatory procedures between injectable and oral vaccines can be attributed to a number of factors. In general, the administration process of injection-type vaccines, which involves direct introduction into the bloodstream, requires a larger sample size in clinical trials to provide sufficient data for efficacy and safety.

Injectable vaccines may contain live viruses or bacteria in a condensed solution, which requires specialized facilities and/or equipment for dilution to prevent contamination and ensure proper handling of the diluted vaccines [27].

There is an additional cost for injection-type vaccines to be distributed into single-dose vials or syringes to ensure consistent dosing and to prevent contamination. The thermostability of a vaccine is critical in ensuring its effectiveness and safety [107]. Most vaccines require a cold-supply chain to maintain their potency during transportation, which may increase the cost of the vaccines by 80% [108]. Unlike oral vaccines, which are generally stored at room temperature, injectable vaccines may require specific refrigeration storage conditions to maintain their stability and efficacy, hindering the feasibility of their distribution in mobile locations [109]. For the COVID-19 vaccines, Pfizer-BioNTech and Moderna must be stored at −80 °C and −20 °C, respectively, and have a 6-month shelf life. When the above vaccines were stored at 2–8 °C, the shelf life dramatically decreased to 30 days [110]. However, oral vaccines maintain high stability at room temperature for months [60].

Clinical trials and regulatory procedures for oral vaccines may incur lower costs, due to their less complex administration process. The manufacturing costs of oral vaccines are generally lower than those of injectable vaccines, as injectable vaccines typically require more complex production processes, including the purification and formulation of the vaccine antigens [111]. However, it is essential to remember that the actual cost of each type of vaccine is subject to the design of the vaccine and its clinical trial programs.

## 5. Challenges of Oral Vaccines

Many challenges need to be overcome in order to address infectious diseases by using oral vaccines. First of all, the vaccines must tolerate the extreme environments of the highly acidic stomach, a wide range of pH values throughout the GI tract, and the presence of proteolytic enzymes that can break down proteins, without a reduction in potency [112]. While numerous immune adjuvants have been utilized in injectable vaccines to boost immune activity, there are a limited number of mucosal adjuvants available for oral immunization. This is because the majority of adjuvants intended for injection are unable to withstand the gastrointestinal mucosal environment. Novel oral vaccine adjuvants which are safe and resistant to harsh environments are required [27].

Secondly, similar to some injectable vaccines, many oral vaccines require multiple doses to elicit a sustained immune response, which makes their use more challenging for people with limited access to healthcare services [113].

Thirdly, as the antigen level in oral vaccines is generally lower than in injectable vaccines, the immune response elicited by oral vaccines is lower. A higher amount of antigen is needed to trigger an immune response compared to typical injection vaccines. Larger doses increase the possibility of inducing tolerance instead of prompting a protective response [27]. Individuals in sensitive groups with a weakened immune system or other underlying health issues may give a substandard response [114].

Finally, the efficacy of oral vaccines can be affected by the gut-associated microbiome, which has an intimate symbiotic relationship with the host. Gut-associated microbiomes regulate the local immune response and can reduce the ability of oral vaccines to trigger the designated immune response [28,115].

Although significant efforts in research and development are required to overcome the above obstacles, it is a worthwhile pursuit when considering the potential benefits of oral vaccines, such as their easier administration, reduced costs, and increased accessibility. Bacillus subtilis (*B. subtilis*), yeast-based, and nanoparticle-based oral vaccines are potential candidates for partially overcoming the above-mentioned challenges. Numerous studies have utilized modified B. subtilis, yeast, and nanoparticles in oral vaccine development [60,116,117]. The budding yeast and *B. subtills* spore is able to survive the extreme environment of the gastrointestinal tract [118,119] and stimulate immunity in the human body [120,121]. Chitosan nanoparticle vaccines are resistant to the simulated gastrointestinal environment, and the antigen is stable under enzyme degradation. In addition, the immune response was shown to be induced in mice when fed the chitosan nanoparticle vaccine [122]. Therefore, B. subtilis, yeast, and nanoparticles are ideal future vaccine vectors for eliciting the desired immune response and for vaccine delivery.

## 6. Recent Development of Oral Vaccines

In recent years, oral vaccines have become a topic of interest as they offer a convenient and accessible alternative to traditional injectable vaccines [123,124]. The objective of current oral vaccine development is to enhance antigen delivery to the GALT, thereby triggering a robust immune response [27]. The advancement in oral vaccine technology involves the utilization of innovative platforms, such as recombinant bacteria and viruses, as well as nanoparticle-based delivery systems. The purpose of these platforms is to overcome the barriers to traditional oral vaccines, which include low antigen stability and insufficient antigen delivery to the GALT [125]. These developments are promising for the prevention and control of infectious diseases, offering a convenient and accessible option, particularly in resource-limited settings.

### 6.1. Bacillus-Subtilis-Based Oral Vaccines

*Bacillus subtilis (B. subtilis)* is generally recognized as safe (GRAS) by the FDA, due to its safety profile and a long history of use as a probiotic and food additive [126]. Certain strains of the species are employed as a well-known platform for heterologous protein expression, because of their fast growth rate, inexpensive genetic engineering in molecular cloning, and efficient utilization of codons [127]. Furthermore, the highly stable recombinant *B. subtilis* plasmids are ideal for the large-scale production of recombinant proteins [128].

**Figure 3:**
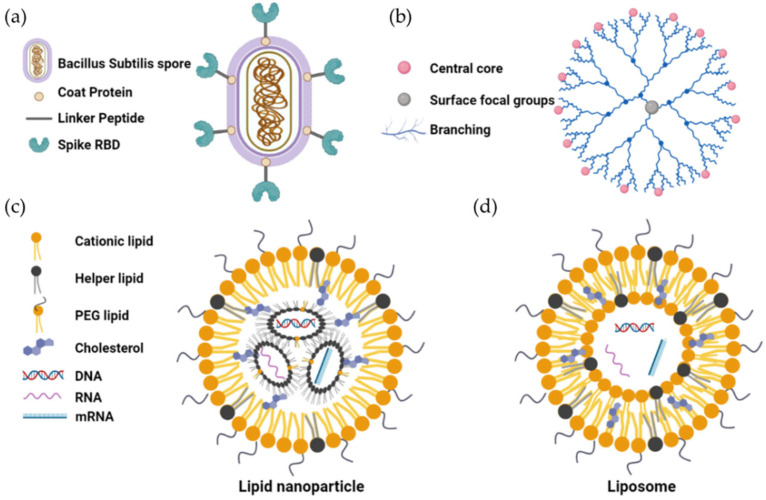
The structure of the *Bacillus subtilis* vaccine (**a**), dendrimer vaccine (**b**), liposome (**c**), and lipid nanoparticle vaccine (**d**). (**b**) Dendrimers are water-soluble nanoscale macromolecules with a highly branched, tree-like structure. Their size ranges from 1 nm to 10 nm. Lipid nanoparticles (**c**) and liposomes (**d**) are vesicle-like supramolecular structures of 50–500 nm size. Both liposomes and lipid nanoparticles can be designed to be soluble in water or oil, depending on the composition of the lipid bilayer.

The resistance to environmental stress of *B. subtilis* spores make them an option for vaccine delivery [118]. The spore is capable of persisting in the small intestine for extended periods of time, which could facilitate the induction of mucosal immunity in the GALT via a recombinant antigen displayed on the surface of the spore [129,130]. The potential of B. subtilis spores in oral vaccines for various diseases, such as SARS-CoV-2 [60] (Figure 3), *Mycobacterium tuberculosis*, and *Clostridium tetani*, has been explored and studied, and the results of the antibody levels and effective neutralization of disease-causing antigens were positive [60].

In studies of SARS-CoV-2 [60] (Figure 3), spike proteins of SARS-CoV-2 were expressed and displayed on the surface of *B. subtilis* spores. Clinical studies have shown that the oral administration of three doses of these *B. subtilis* spores resulted in a significant elevation of both IgG and IgA antibodies against the spike proteins. On the other hand, it was shown that the serum collected from volunteers was able to neutralize the pseudovirus expressing the spike protein from both wild-type and D614G variants of SARS-CoV-2 [60].

### 6.2. Nanoparticle-Based Delivery System for Oral Vaccines

Nanotechnology is a novel and attractive platform for oral vaccine delivery [131,132]. The use of nanoparticles as vaccine-delivery vehicles greatly enhances the stability of antigens and the efficacy of immunity induction. The efficiency of antigen delivery is significantly influenced by various factors, including particle size, charge, surface functional groups, and shape [117]. Recently, nanoparticles such as liposomes, lipid nanoparticles, dendrimers, and inorganic nanoparticles are the most widely studied nanoparticles for oral vaccine delivery [133,134].

Both liposomes and lipid nanoparticles are nanoscale vesicle-like supramolecular structures, consisting of lipid molecules and/or cholesterols and other organic molecules. Liposomes are spherical and have a lipid bilayer that encapsulates a mixture of core ingredients, including proteins, RNA, or other biomolecules [135]. Lipid nanoparticles are even smaller than liposomes and can more easily penetrate the cell. Both types of nanoparticles are flexible and effective for the protection and encapsulation of antigens from degradation by stomach acid in the gut, thereby increasing their immunogenicity [135]. An oral vaccine delivered by liposomes against rotavirus demonstrated safety and effectiveness in the induction of an immune response [136]. PLGA (Poly(lactic-co-glycolic acid)) polymeric nanoparticles are composed of biodegradable and biocompatible polymers with the ability to encapsulate antigens and protect them from degradation in the gut [137]. These nanoparticles can be designed to target specific cells or tissues and increase vaccine efficacy by coupling with adjuvants or other immune-stimulating molecules. A PLGA nanoparticle-based oral vaccine coated with M cell homing receptors was able to elicit protective immune responses in vivo [138]. The acid-resistant PLGA nanoparticle oral vaccine, designed against Helicobacter pylori infection, induced T-cell responses and antibody production in mice after oral vaccination [139].

Dendrimers are nano-size, highly branched macromolecules that can couple with antigens and adjuvants for the induction of an immune response [140] (Figure 3). In addition, specific ligands can be assigned to dendrimers to bind to corresponding receptors on the surface of immune cells for specific cell or tissue delivery. Oral vaccines delivered by dendrimers against Salmonella, Shigella, and *Helicobacter pylori (H. pylori)* have been shown to elicit significant immunogenicity and efficacy [141]. However, there are still challenges to the use of dendrimers for oral vaccine delivery to be addressed, including toxicity and production cost.

Inorganic nanoparticles are small particles made of non-organic materials, such as metals, metal oxides, or semiconductors. They possess unique properties due to their small size and high surface-to-volume ratio [142]. Carbon nanoparticles and silica nanoparticles have been extensively utilized in the development of oral vaccines. A carbon nanoparticle-based oral vaccine produced a similar level of IgG as the intramuscular injection route. Additionally, mucosal IgA was detected, indicating an effective immune response had been initiated [143]. When administered orally, a silica nanoparticle vaccine elicited mucosal and systemic immune responses in vivo [144].

Nanoparticle-based oral vaccines offer several advantages compared to traditional vaccine delivery methods. Nanoparticles provide a protective coating for the vaccine components, preventing degradation in the harsh gastrointestinal environment. In addition, the bioavailability of vaccine antigens is enhanced by facilitating absorption through the intestinal epithelium. Currently, challenges such as toxicity and large-scale production are major hurdles to their application in oral vaccine delivery.

Although nanoparticle-based oral vaccines are still in the early stages, they have shown promise in animal tests and the early phases of clinical trials [117]. Nanoparticle-based oral vaccine delivery systems have the potential to revolutionize the field of vaccine development.

## 7. Conclusions

Oral vaccines demonstrate immense potential for combating contagious diseases worldwide. The advantages of this immunization approach are evident in the simplified delivery method, enhanced adherence, and cost-effectiveness. Given difficulties such as instability and low potency in poverty-stricken nations, advanced innovations in oral vaccines, such as the utilization of *Bacillus subtilis* and nanotechnologies, look likely to combat these difficulties and enhance their efficacy. Ongoing studies and medical trials are necessary to fully unlock the ability of oral vaccines to fight infectious diseases. With advanced technology and dedication to health worldwide, these immunizations have the capacity to surpass limitations and lay the foundation for a brighter immunization future.

## 8. Patents

Patents resulting from the work reported in this manuscript were filed: Chinese (patent number: 202111143384.9), Hong Kong (patent number: 32021042343.2), and PCT.

## Figures and Tables

**Table 1:** Comparison between oral vaccines and injection vaccines.

	Oral Vaccines	Injection Vaccines
Administration Route	Oral (non-invasive)	Mostly intramuscular or subcutaneous (invasive)
The Site Producing a Protective Response	Mucosal and systemic	Systemic
Secretory Immunoglobulin	Mainly IgA	IgG
Cost	Relatively low	High
Manufacturing Procedure	Relatively simple and do not require an extensive purification process	Extensive purification process needed with higher standard aseptic equipment
Distribution	Easy to distribute	Professional healthcare workers and specific locations required
Dosage	Higher doses due to degradation in the stomach and intestine	Relatively accurate

**Table 2:** Licensed oral vaccines.

Disease	Vaccine Name	Antigens	References
Polio	Polio Sabin	Live-attenuated Sabin strains 1,2,3	[11,31]
Cholera	Dukoral	Inactivated strains (types) of *V. cholerae* serotype O1 and recombinant cholera toxin B subunit (rCTB)	[32,33]
	Vaxchora	Weakened cholera bacterium *Vibrio cholerae* (serogroup O1)	[34,35]
Rotavirus	RotaTeq	Live rotavirus strains containing antigen G1, G2, G3, G4 and P1(8)	[36,37]
	Rotarix	Weakened human rotavirus RIX4414 strain	[38,39]
Typhoid fever	Vivotif	Live attenuated strain Salmonella typhi Ty21a (1,2)	[40,41]

## Data Availability

No new data were created or analyzed in this study. Data sharing is not applicable to this article.
